# Blind Image Quality Assessment of Natural Scenes Based on Entropy Differences in the DCT Domain

**DOI:** 10.3390/e20110885

**Published:** 2018-11-17

**Authors:** Xiaohan Yang, Fan Li, Wei Zhang, Lijun He

**Affiliations:** 1 School of Electronic and Information Engineering, Xi’an Jiaotong University, Xi’an 710049, China; 2 The State Key Laboratory of Integrated Services Networks, Xidian University, Xi’an 710071, China

**Keywords:** blind image quality assessment (BIQA), information entropy, natural scene statistics (NSS), Weibull statistics, discrete cosine transform (DCT)

## Abstract

Blind/no-reference image quality assessment is performed to accurately evaluate the perceptual quality of a distorted image without prior information from a reference image. In this paper, an effective blind image quality assessment approach based on entropy differences in the discrete cosine transform domain for natural images is proposed. Information entropy is an effective measure of the amount of information in an image. We find the discrete cosine transform coefficient distribution of distorted natural images shows a pulse-shape phenomenon, which directly affects the differences of entropy. Then, a Weibull model is used to fit the distributions of natural and distorted images. This is because the Weibull model sufficiently approximates the pulse-shape phenomenon as well as the sharp-peak and heavy-tail phenomena of natural scene statistics rules. Four features that are related to entropy differences and human visual system are extracted from the Weibull model for three scaling images. Image quality is assessed by the support vector regression method based on the extracted features. This blind Weibull statistics algorithm is thoroughly evaluated using three widely used databases: LIVE, TID2008, and CSIQ. The experimental results show that the performance of the proposed blind Weibull statistics method is highly consistent with that of human visual perception and greater than that of the state-of-the-art blind and full-reference image quality assessment methods in most cases.

## 1. Introduction

The human visual system (HVS) is important for perceiving the world. As an important medium of information transmission and communication, images play an increasingly vital role in human life. Since distortions can be introduced during image acquisition, compression, transmission and storage, the image quality assessment (IQA) method is widely studied for evaluating the influence of various distortions on perceived image quality [1,2].

In principal, subjective assessment is the most reliable way to evaluate the visual quality of images. However, this method is time-consuming, expensive, and impossible to implement in real-world systems. Therefore, objective assessment of image quality has gained growing attention in recent years. Depending on to what extent a reference image is used for quality assessment, existing objective IQA methods can be classified into three categories: full-reference (FR), reduced-reference (RR) and no-reference/blind (NR/B) methods. Accessing all or part of the reference image information is unrealistic in many circumstances [3,4,5,6,7,8], hence it has become increasingly important to develop effective blind IQA (BIQA) methods.

Many NR IQA metrics focus on assessing a specific type of visual artifact, such as blockiness artifacts [9], blur distortion [10], ringing distortion [11] and contrast distortion [12]. The main limitation is that the distortion type must be known in advance. However, generic NR-IQA metrics have recently become a research hotspot because of their general applicability.

According to the dependency on human opinion scores, the generic NR approaches can be roughly divided into two categories [13]: distance-based methods and learning-based methods. Distance-based methods express the image distortion as a simple distance between the model statistics of the pristine image and those of the distorted image [14,15,16]. For example, Saha et al. [16] proposed a completely training-free model based on the scale invariance of natural images.

Learning-based methods have attracted increasing attention with the development of artificial intelligence. The basic strategy is to learn a regression model that maps the image features directly to a quality score. Various regression methods, including support vector regression (SVR) [17], neural network [18,19,20], random forest regression [21] and deep learning framework [22,23], are widely used for model learning. More importantly, after pre-processing of image [24], image features, which are extracted for model learning, are directly related to the accuracy of the IQA. The codebook-based method [25] aims at extracting Gabor filter-based local features, which describe changes of texture information. Moreover, NSS-based methods are also widely used to extract features [26,27,28,29,30,31,32,33]. In [26,27], these methods use the difference of NSS histogram of natural and distorted images to extract image features. In [28,29,30,31,32,33], they aim to establish NSS model to extract features. The Laplace model [28], the Generalized Gaussian distribution (GGD) model [29,32,33], the generalized gamma model [30] and Gaussian scale mixture model [31] are widely used as NSS model to extract features in different domains. In addition, Ghadiyaram et al. combined histogram features and NSS model features to achieve good-quality predictions on authentically distorted images [34].

NSS model-based methods have achieved promising results. The NSS model assumes that natural images share certain statistical regularities and various distortions may change these statistics. Therefore, the NSS model is capable of fitting statistics of natural and distorted images. The GGD model is a typical NSS model that is widely studied and applied. The GGD model in the DCT domain is able to follow the heavy-tail and sharp-peak characteristics of natural images. By using the GGD features, the distorted image quality can be estimated. However, the GGD model has some shortcomings in fitting the statistics of distorted images because the distribution of DCT coefficients shows a pulse-shape phenomenon for distorted images, which is described as the rapid increase of discontinuity. The discontinuity is derived from the differences between the high- and low-frequency coefficients of distorted images. Thus, the pulse-shape phenomenon cannot be fitted by the GGD model, which leads to inaccurate quality assessments.

In this paper, an effective blind IQA approach of natural scenes related to entropy differences is developed in the DCT domain. The differences of entropy can be described by probability distribution in distorted images. We find the DCT coefficients’ distribution of distorted images shows a pulse-shape phenomenon in addition to the heavy-tail and sharp-peak phenomena. Since the pulse-shape phenomenon is often neglected in NSS model, image structure cannot be fully presented by image entropy. Therefore, the performance of the IQA methods based on such NSS model can be affected to some extent. To this end, the Weibull model is proposed in this paper to overcome the under-fit caused by the pulse shape phenomenon of traditional GGD model. Furthermore, we prove that the Weibull model correlates well with the human visual perception. Based on the Weibull model, corresponding features are extracted in different scales and the prediction model is derived using the SVR method. Experimental results show that the Weibull statistics (BWS) method consistently outperforms the state-of-the-art NR and FR IQA methods over different image databases. Moreover, the BWS method is a generic image quality algorithm, which is applicable to multiple distortion types.

The novelty of our work lies in that we find the pulse-shape phenomenon when using existing GGD model to characterize image distortions. Then, we propose a useful Weibull model to overcome under-fit the pulse-shape phenomenon and extract features related to visual perception from the Weibull model to evaluated image quality. Furthermore, the proposed method has the advantage of high prediction accuracy and high generalization ability.

The rest of the paper is organized as follows. Section 2 presents the NSS model based on entropy differences. Section 3 presents the proposed BWS algorithm in details. Section 4 evaluates the performance of the BWS algorithm from various aspects. Section 5 concludes the paper.

## 2. NSS Model Based on Entropy Differences

Information entropy indicates the amount of information contained within an image and the changes of entropy are highly sensitive to the degrees and types of image distortions. Our method utilizes distribution difference of DCT coefficients that directly affects entropy changes to assess image quality. As reported in the literature [35,36], natural images exhibit specific statistical regularities in spatial and frequency domains and are highly structured, and the statistical distribution remain approximately the same under scale and content changes. The characteristics of natural images are essential for IQA.

### 2.1. Under-Fitting Effect of GGD

The GGD model, as a typical NSS model, is widely used to fit distribution of AC coefficients in the DCT domain for natural images [29], and it can well simulate non-Gaussian behaviors, including sharp-peak and heavy-tail phenomena of the distribution of AC coefficients of natural images [35,36,37].

**Figure 1:**
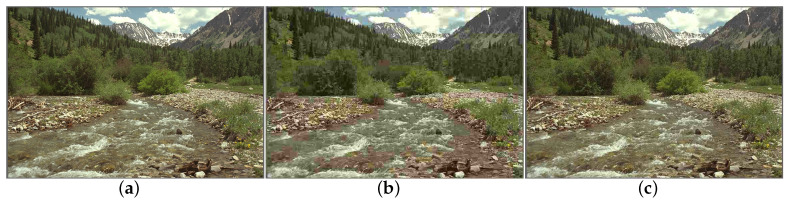
One natural image (“stream”) and two of its JPEG-compressed versions in the LIVE database: (**a**) image with DMOS = 0; (**b**) image with DMOS = 63.649; and (**c**) image with DMOS = 29.739.

**Figure 2:**
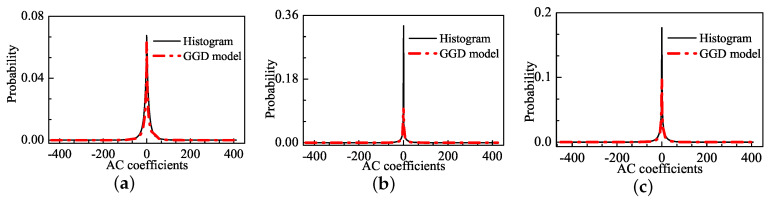
The distribution of AC coefficients for corresponding images and the GGD fitting curves: (**a**) image with DMOS = 0; (**b**) image with DMOS = 63.649; and (**c**) image with DMOS = 29.739.

Figure 1 shows a natural image (i.e., “stream”) included in the LIVE database [38], and two of its JPEG-compressed versions. The subjective qualities are scored by the Difference Mean Opinion Score (DMOS), which returned values of 0, 63.649 and 29.739 for the three images. A larger DMOS indicates lower visual quality. Figure 2 shows the distribution of AC coefficients for the corresponding images in Figure 1 and the GGD fitting curve. As shown in Figure 2a, the GGD model is capable of fitting the natural image, especially the sharp-peak and heavy-tail phenomena. For the distorted images, however, distinct deviations occur around the peak, as shown in Figure 2b,c. The underlying reason for the misfits is that the structure information of the distorted images has been changed including smoothness, texture, edge information. The number of the AC coefficients in the value of zero is increased rapidly, which triggers the pulse-shape. The pulse-shape phenomenon enhances along the increase of the distortion level. Thus, the GGD model fails at simulating the pulse-shape phenomenon, and it under-fits the distorted image distribution.

**Weibull** **Distribution Model**

**Figure 3:**
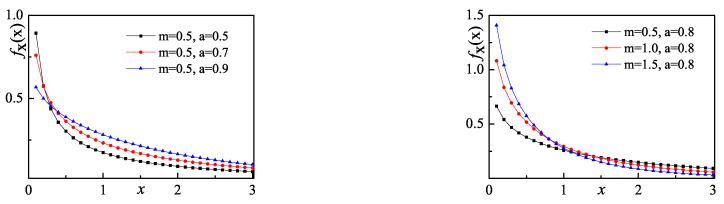
The change of Weibull distribution in different parameter settings.

To well fit the distribution of AC coefficients for a distorted image, the Weibull distribution model is employed, which is given by [39]:

(1) $$f_{X}\left(x\right)=\frac{a}{m}{\left(\frac{x}{m}\right)}^{a-1}\exp\left[-{\left(\frac{x}{m}\right)}^{a}\right]\;x>0$$

where *a* and *m* are the shape parameter and the scale parameter. The family of Weibull distributions includes the Exponential distribution (a=1) and Rayleigh distribution (a=2). When a<1, $f_{X}\left(x\right)$ approaches infinity as *x* approaches zero. The characteristic can be used to describe the pulse-shape phenomenon for the distribution of AC coefficients. Figure 3 shows the change of Weibull distribution in different parameter settings. When *m* is fixed and *a* is less than 1, a larger shape parameter *a* corresponds to a slower change of $f_{X}\left(x\right)$. When *a* is fixed, a larger scale parameter *m* corresponds to a faster change of $f_{X}\left(x\right)$.

**Figure 4:**
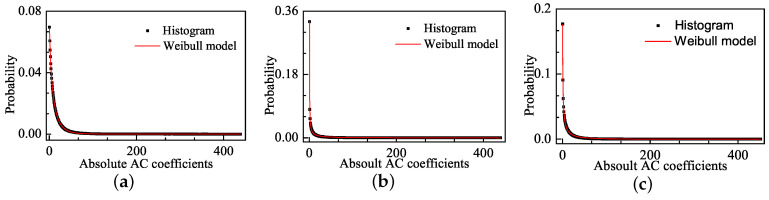
The distribution of absolute AC coefficients corresponding images and the Weibull fitting curves: (**a**) image with DMOS = 0; (**b**) image with DMOS = 63.649; and (**c**) image with DMOS = 29.739.

Figure 4 shows the distribution of the absolute values of AC coefficients for the corresponding images in Figure 1 and the fitting curves of the Weibull model. The Weibull model well fits the pulse-shape in addition to the sharp-peak and heavy-tail phenomena. The faster the Weibull distribution changes, the larger the distortion is. We use the Mean Square Error (MSE) to express the fitting error:

(2) $$MSE=\frac{1}{n}\sum_{n=1}^{n}{\left(P_{i}-Q_{i}\right)}^{2}$$

where $P_{i}$ is the value of the histogram for the absolute values of AC coefficients, and $Q_{i}$ is the statistical distribution density of the fitting functions. The MSE values for Weibull model are $6.07\times 10^{-6}$ and $3.38\times 10^{-7}$ in Figure 4b,c, respectively, and these values are much lower than the MSE values of the GGD model, which are $1.1\times 10^{-4}$ and $3.36\times 10^{-5}$ in Figure 2b,c, respectively.

**Figure 5:**
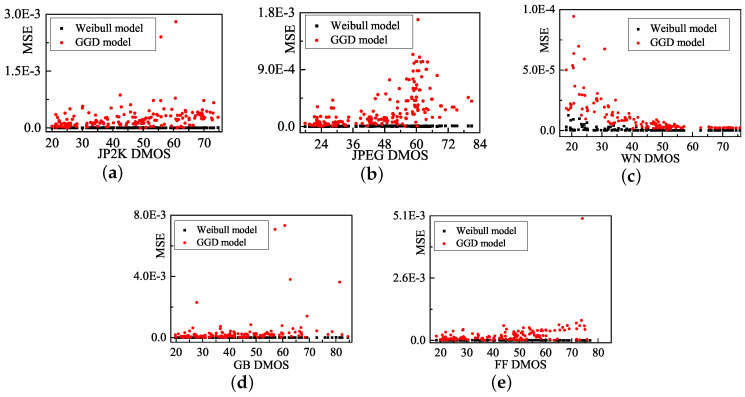
Fitting error of GGD model and Weibull model in LIVE database.

We also develop a Weibull model that fits five representative distortion types in the LIVE database: JP2K compression (JP2K), JPEG compression (JPEG), white noise (WN), Gaussian blur (GB), and fast-fading (FF) channel distortions [38]. Figure 5 presents the MSE comparison of two models for each distortion type images. Table 1 lists the average MSE of each distortion type. The fitting error of the Weibull model is obviously smaller than that of the GGD model. Therefore, the Weibull model is employed to evaluating the image quality in this paper.

## 3. Proposed BWS Method

**Figure 6:**
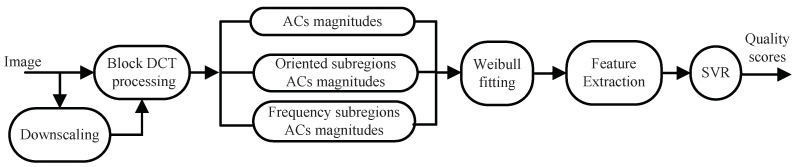
Framework of the BWS algorithm.

In this section, we describe the proposed BWS method in detail. The framework is illustrated in Figure 6. First, block DCT processing is applied to images of different scales. The goal is not only to conform to the decomposition process of local information in the HVS [40] but also to reflect the image structure correlation [4]. In the DCT domain, the magnitudes of the block AC coefficients and those in the orientation and frequency sub-regions are extracted to describe HVS characteristics. Then, a Weibull model is employed to fit these values. From the Weibull model, the following four perceptive features are extracted: the shape-scale parameter feature, the coefficient of variation feature, the frequency sub-band feature, and the directional sub-band feature. Finally, by using the SVR learning method, the relationship between the image features and subjective quality scores in high-dimensional space is obtained.

One advantage of this approach is that we consider only the change of luminance information in the BWS algorithm because neuroscience research shows that the HVS is highly sensitive to changes in image luminance information [37]. In addition, DCT processing is useful in IQA. The presentation of features can be enhanced by treating different frequency components with different distortion levels. The computational convenience is another advantage [41].

### 3.1. Relationship Between HVS and Perceptual Features

IQA modeling must be able to satisfy human perceptual requirements, which are closely related to HVS. Designing an HVS-based model for directly predicting image quality is infeasible because of the complexity. Therefore, in this paper, the features related to the HVS are extracted from the Weibull model and used to predict perceptual image quality scores by a learning method.

The visual perception system has been shown to be highly hierarchical [42]. Visual properties are processed in areas V1 and V2 of the primate neocortex, which occupies a large region of the visual cortex. V1 is the first visual perception cortical area, and the neurons in V1 can achieve succinct descriptions of images in terms of the local structural information pertaining to the spatial position, orientation, and frequency [43]. Area V2 is also a major visual processing area in the visual cortex [44], and the neurons in V2 have the property of the scale invariance [45]. Therefore, our proposed model is related to the properties of the HVS.

### 3.2. Shape-Scale Parameter Feature

Before extracting features, we divide the image into 5×5 blocks with a two-pixel overlap between adjacent blocks to remove the redundancy of image blocks and better reflect the correlation information among blocks. For each block, DCT is performed to extract the absolute AC coefficients. Then, the Weibull model is used to fit the magnitudes of the AC coefficients of each block. Theories in fragmentation posit that the scale parameter *m* and shape parameter *a* in the Weibull distribution are strongly correlated with brain responses. The experiments on brain responses showed that *a* and *m* explain up to 71 % of variance of the early electroencephalogram signal [39]. These parameters can also be estimated from the outputs of X-cells and Y-cells [46]. In addition, the two parameters can accurately describe the image structure correlation because a difference in the image distribution of quality degradation, which depends on the image structural information, results in a different shape of the Weibull distribution, thereby resulting in different values of *a* and *m*. In other words, the response of the brain to external image signals is highly correlated with the parameters *a* and *m* of the Weibull distribution. Thus, we defined the shape-scale parameter feature $\zeta ={\left(\left.1/m\right.\right)}^{a}$. The parameters that directly determine the form of the Weibull distribution are *a* and ${\left(\left.1/m\right.\right)}^{a}$, as shown in Equation (3). The Equation (3) is the deformation of Weibull distribution. Because the human subjects are viewing natural images that are correlated with *a* and *m*, considering only the influence of *a* while ignoring the effect of *m* on the Weibull distribution does not produce accurate results. Therefore, we chose ${\left(\left.1/m\right.\right)}^{a}$ as feature ζ for assessing image quality. The advantage of this feature is that it provides an intuitive expression of the Weibull equation as well as a monotonic function of distortion, which can be used to represent the levels of distortion in images.

(3) $$f_{X}\left(x\right)=a\left({\frac{1}{m}}^{a}\right)x^{a-1}\exp\left[-{\left(\frac{1}{m}\right)}^{a}x^{a}\right]\;x>0$$

The efficiency of the features is verified in the LIVE database. We extracted the average value of the highest 10% and 100% (all block coefficients) of the shape-scale parameter features (ζ) in all blocks of the image. These two percentages correspond to image distortion of the local worst regions and the global regions, respectively. It may be inappropriate to only focus on distortion of local worst regions or the overall regions [47,48,49]. Thus, it is necessary to combine the local distortion with the global distortion.

Table 2 shows the Spearman Rank Order Correlation Coefficient (SROCC) values between the DMOS scores and the average values of the highest 10% of ζ as well as the DMOS values and the average values of the 100% of ζ in LIVE database. The SROCC value is larger than 0.7, which indicates a significant correlation with the subjective scores [50]. Therefore, these features can be effectively used as perceptual features for IQA.

### 3.3. Coefficient of Variation Feature

Natural images are known to be highly correlated [36,37]. The correlation can be affected by distortions in different forms. In the DCT domain, distortions change the distribution of the AC coefficients. For JP2K, JPEG, GB, and FF [38], the distortion increases the differences among low-, middle- and high-frequency information. Then, the standard deviation becomes larger under the unit mean than that in the natural image. Thus, the large variation of the standard deviation represents a large distortion. In contrast, for WN distortions, the increased random noise causes high-frequency information to increase rapidly, thereby reducing the differences among different frequency information. Thus, a small variation of the standard deviation corresponds to a large distortion.

Therefore, we define the coefficient of variation feature ξ, which describes the variation of the standard deviation under the unit mean as follows:

(4) $$\xi =\frac{\sigma_{X}}{\mu_{X}}=\sqrt{\frac{\Gamma (1+\frac{2}{a})}{\Gamma^{2}\left(1+\frac{1}{a}\right)}-1}$$

where the mean $\mu_{X}$ and variance $\sigma_{X}^{2}$ of the Weibull model can be obtained as follows:

(5) $$\mu_{X}=\int_{0}^{\infty }xf_{X}\left(x\right)\;dx\;=m\Gamma \left(1+\frac{1}{a}\right)$$

(6) $$\sigma_{X}^{2}=\int_{0}^{\infty }x^{2}f_{X}\left(x\right)\;dx\;-\mu^{2}=m^{2}\Gamma \left(1+\frac{2}{a}\right)-m^{2}\Gamma^{2}\left(1+\frac{1}{a}\right)$$

where Γ denotes the gamma function. This parameter is defined as follows:

(7) $$\Gamma \left(z\right)=\int_{0}^{\infty }t^{z-1}e^{-t}\;dt\;$$

We calculated the average value of the highest 10% of ξ and the average value of 100% of ξ in all blocks across the image. Table 3 shows the SROCC values, which are verified in the LIVE database. The correlation is also significant in most distortion types. Thus, the features can be used to assess image quality.

### 3.4. Frequency Sub-Band Feature

Natural images are highly structured in the frequency domain. Image distortions often modify the local spectral properties of an image so that these properties are dissimilar to those of in natural images [29]. For JP2K, JPEG, GB, and FF distortions, distortions trigger a rapid increase of differences among the coefficients of variation of the frequency sub-bands coefficients. A large difference represents a large distortion. However, with the WN distortion type, the opposite change trend is observed.

To measure this difference, we defined the frequency sub-band feature *f*. According to the method in [29], we divided each 5×5 image block into three different frequency sub-bands, as shown in Table 4. Then, the Weibull fit was obtained for each of the sub-regions, and the coefficient of variation $\xi_{f}$ was calculated using Equation (4) in the three sub-bands. Finally, the variance of $\xi_{f}$ was calculated as the frequency sub-band feature *f*.

The feature was pooled by calculating the average value of the highest 10% of *f*, and the average value of 100% of *f* in all blocks across the image in the LIVE database. In Table 5, we report how well the features are correlated with the subjective scores. The SROCC is clearly related to the subjective scores, which means that these features can be used to describe subjective perception.

### 3.5. Directional Sub-Band Feature

The HVS also has different sensitivities to sub-bands in different directions [40]. Image distortion often changes the correlation information of sub-bands in different directions, which makes the HVS highly sensitive to this change. For the JP2K, JPEG, GB, and FF distortion types, distortions modify the inconsistencies among coefficients of variation in sub-bands in different directions. A large inconsistency reflects a large distortion. Note that this effect has a reverse relationship to the WN distortion.

Therefore, this inconsistency can be described by the orientation sub-band feature $S_{o}$. We divided sub-bands into three different orientations for each block, as shown in Table 6. This decomposition approach is similar to the approach in [29]. Then, the Weibull model was fitted to the absolute AC coefficients within each shaded region in the block. The coefficient of variation $\xi_{o}$ was also calculated, as shown in Equation (4), in three directional sub-bands. Finally, the directional sub-band feature $S_{o}$ can be obtained from the variance of $\xi_{o}$.

The average values of the highest 10% and 100% of $S_{o}$ for all blocks across images were collected. We report the SROCC values between DMOS scores and features in Table 7 and demonstrate an obvious correlation with human perception.

### 3.6. Multi-Scale Feature Extraction

Previous research has demonstrated that the incorporation of multi-scale information can enhance the prediction accuracy [5]. The statistical properties of a natural image are the same at different scales, whereas distortions affect the image structure across different scales. The perception of image details depends on the image resolution, the distance from the image plane to the observer and the acuity of the observer’s system [40]. A multi-scale evaluation accounts for these variable factors. Therefore, we extracted 24 perceptual features across three scales. In addition to the original-scale image, the second-scale image was constructed by low-pass filtering and down-sampling the original image by a factor of two. Then, the third-scale image was obtained in the same way from the second-scale image. As listed in Table 8, each scale includes eight features. The extraction process is as described in Section 3.2, Section 3.3, Section 3.4 and Section 3.5.

### 3.7. Prediction Model

After extracting features in three scales, we learned the relationship between image features and subjective scores. In the literature, SVR is widely adopted as the mapping function for learning this relationship [51,52]. Considering a set of training data $\left\{\left(x_{1},y_{1}\right),\cdots ,\left(x_{l},y_{l}\right)\right\}$, where $x_{i}\in R^{n}$ is the extracted image feature and $y_{i}$ is the corresponding DMOS, a regression function can be learned to map the feature to the quality score, i.e., $y_{i}=SVR\left(x_{i}\right)$. We used the LIBSVM package [53] to implement the SVR with a Radial Basis Function (RBF) kernel in our metric. Once the regression model was learned, we could use it to estimate the perceptual quality of any input image.

## 4. Experiments and Results

### 4.1. Experimental Setup

The performance of the blind IQA algorithms was validated using subjective image quality databases, where each image is associated with a human score (e.g., a (Difference) Mean Opinion Scores (DMOS/MOS)). The performance describes how well the objective metric is correlated with human ratings. Several subjective image quality evaluation databases have been established. We employed three widely used databases, namely, the LIVE database [38], the TID2008 database [54] and the CSIQ database [55], in our research. These three databases are summarized as follows:

(1) The LIVE database includes 29 reference images and 779 distorted images corrupted by five types of distortions: JP2K, JPEG, WN, GB and FF. Subjective quality scores are provided in the form of DMOS ranging from 0 to 100. Each of the distorted images is associated with a DMOS, representing the subjective quality of this image.
(2) The TID2008 database covers 17 distortion types, each of which consists of 100 distorted versions from 25 reference images. Subjective quality scores are provided for each image in the form of MOS, ranging from 0 to 9. Note that there is one artificial image and its distortion images in the TID2008 database. We discarded these images when evaluating the performance of the BWS method because the proposed method was designed to evaluate the quality of natural scenes. In addition, we mainly considered the subsets with JP2K, JPEG, WN and GB distortion types that appear in LIVE database. These four distortion types are also the most commonly encountered distortions in practical applications.
(3) The CSIQ database consists of 30 reference images and 866 distorted images corrupted by six types of distortions: JPEG, JP2K, WN, GB, pink Gaussian noise and global contrast decrements. Each distorted image has five different distortion levels. Subjective quality scores are provided in the form of DMOS ranging from 0 to 1. Similarly, we mainly considered the same four distortion types as included the LIVE database.

To evaluate the performance of the BWS algorithm, two correlation coefficients, the Pearson Linear Correlation Coefficient (PLCC) and the SROCC, are used as the criteria. The PLCC measures the prediction accuracy, whereas the SROCC represents the prediction monotonicity. Before calculating the PLCC, the algorithm scores are mapped using a logistic non-linearity as described in [56]. Both the SROCC and PLCC lie in the range [−1,1]. SROCC and PLCC values that are closer to “1” or “−1”correspond to better predictions of this algorithm.

### 4.2. Performance on Individual Databases

First, we evaluated the overall performance of the BWS method and other competing IQA methods, namely, BLIINDS-II [29], DIIVINE [31], BRISQUE [32], BIQI [57], PSNR and SSIM [4], on each database. The first four methods are NR IQA algorithms, and the latter two are FR IQA algorithms.

Because the BWS approach is based on SVR, we randomly divided each image database into two sets: a training set and a testing set. The training set is used to train the prediction model and the testing set is used to test the prediction results. In our experimental setup, 80% of the distorted images for each database are used as the training set and the remaining 20% of the images are used as the testing set. Content does not overlap between these two sets. We repeat the training–testing procedure 1000 times, and the median value of the obtained SROCCs and PLCCs is reported as the final performance of the proposed metric. Meanwhile, we adapted the same experimental setup for comparison algorithms. Although the FR IQA approaches PSNR and SSIM do not require training on a database, for a fair comparison, we also conducted the experiments on the randomly partitioned testing set and recorded the median value of the PLCC and SROCC.

Table 9 shows the performances of different methods on the LIVE, TID2008 and CSIQ databases. For each database, the top two IQA methods are highlighted in bold. For the LIVE database, the overall performance of the proposed BWS method is better than those of the other IQA methods. For the TID2008 database and the CSIQ database, the BWS algorithm outperforms the other NR and FR IQA methods. It is concluded that the BWS algorithm outperforms the other competitors overall on different databases. Although other methods may work well on some databases, they fail to deliver good results on other databases. For example, DIIVINE obtains a good result on the LIVE database but performs poorly on the CSIQ and TID2008 databases.

Moreover, we present weighted-average SROCC and PLCC results of competing IQA methods on all three databases in Table 10. The weight that is assigned to each database depends on the number of distorted images that the database contains [58,59]. BWS still performs best among the IQA methods. Hence, we conclude that the objective scores that are predicted by BWS correlate much more consistently with subjective evaluation than those that are predicted by other IQA metrics.

To determine whether the superiority of the BWS method over its counterparts is statistical significance, we conducted statistical analysis to validate their differences in performance. The hypothesis testing, which was based on the t-test [60], was presented, which measures the equivalence of the mean values of two independent samples. Experiments are conducted by randomly splitting the database into a training set and a testing set and the SROCC values are reported for 1000 training-testing trials. Thus, we applied the t-test between the SROCCs that were generated by each of the two algorithms and tabulated the results in Table 11, Table 12 and Table 13. Each table shows the results of the t-test on each database. A value of “1” in the tables indicates that the row algorithm is statistically superior to the column algorithm, whereas a value of “−1” indicates that the row algorithm is statistically inferior to the column algorithm. A value of “0” indicates that the row and column algorithms are statistically equivalent. From the experimental results in Table 10, Table 11 and Table 12, the BWS method was found to be statistically superior to FR approaches PSNR and SSIM and NR IQA approach BLIINDS-II.

### 4.3. Performance on Individual Distortion Type

In this section, we tested the performances of the proposed BWS method and other competing IQA methods on individual distortion type over the LIVE, TID2008 and CSIQ databases. For NR IQA, we trained on 80% of the distorted images with various distortion types randomly and tested on the remaining 20% of the distorted images with a specific distortion type. The SROCC and PLCC comparisons on each database are illustrated in Table 14 and Table 15.

We observed the advantages of the individual distortion type of the BWS algorithm on each database. For the LIVE database, we clearly found that the proposed metric outperforms other NR metrics in FF. In particular, compared with the BLIINDS-II method, implemented in the same DCT domain, BWS performs better on the JP2K, WN, GB and FF distortion types. For FR metrics, although these methods require the complete information of the reference images, our algorithm still outperforms PSNR on all distortion types and outperforms SSIM on WN and GB distortions. For the TID2008 database, the performance of our method is superior on JP2K distortions relative to other NR metrics. Similarly, the performances on JP2K, WN, and GB distortion are better than those of the BLIINDS-II method. Compared with the FR metric, the performance of the BWS algorithm on the JP2K distortion type is better than those of PSNR and SSIM. For the CSIQ database, our method outperforms the remaining NR metrics on WN and GB distortions. Compared with the BLIINDS-II algorithm, the BWS method consistently performs better. Compared with the FR metric, the performances of BWS on the JPEG and GB types are better than those of PSNR, and on GB and WN the distortion is better than with SSIM. Therefore, we found that the performance of the BWS algorithm is superior on some specific distortion types in each database.

To facilitate a comparison of the effects between BWS method and the other IQA methods, 13 groups of distorted images were considered in the three databases. The best two results of the SROCC and PLCC are highlighted in boldface for the NR IQA methods. We calculated the number of times that each method was ranked in the top two in terms of the SROCC values and PLCC values for each distortion type. For the 13 groups of distorted images in the three databases, the BWS algorithm was ranked in the top two the most times, with 10 times for the SROCC and 11 times for the PLCC. We also report the weighted means and standard deviations (STDs) of competing IQA methods across all distortion groups in Table 16. The BWS has higher average and lower STD across all distortion groups. Hence, the BWS method achieves a consistently better performance on most commonly encountered distortion types.

**Figure 7:**
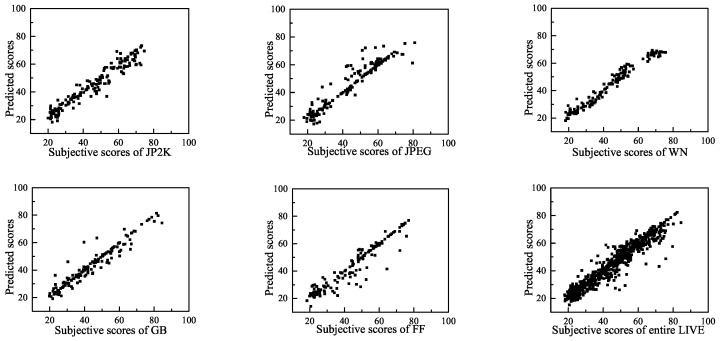
Predicted scores versus subjective scores on LIVE database.

**Figure 8:**
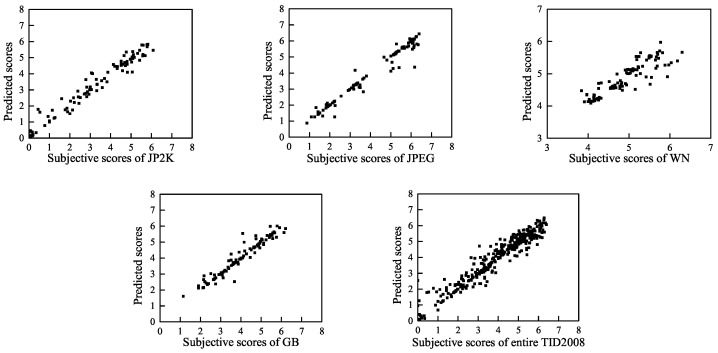
Predicted scores versus subjective scores on TID2008 database.

**Figure 9:**
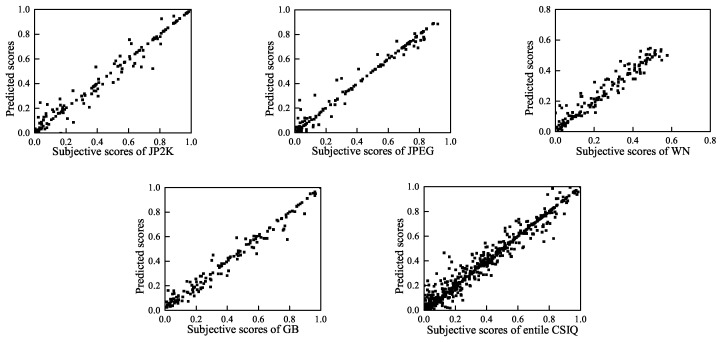
Predicted scores versus subjective scores on CSIQ database.

To visually show the correlation of the BWS method between the predicted quality scores and the subjective scores, we present scatter plots (for each distortion type and for the entire LIVE, TID2008 and CSIQ databases) of the predicted scores and the subjective scores in Figure 7, Figure 8 and Figure 9. These figures show that a strong linear relationship occurs between the predicted scores of BWS and the subjective human ratings, which indicates a high prediction accuracy of the BWS algorithm.

### 4.4. Cross-Database Validation

In our previous experiments, the training samples and test samples were selected from the same database. It is expected that an IQA model that has learned on one image quality database should be able to accurately assess the quality of images in other databases. Therefore, to demonstrate the generality and robustness of the proposed BWS method, the following experiments were conducted. We trained all distorted images from one database to obtain a prediction model and used this model to test the scores of distorted images from other databases. With the three databases, six combinations of training and testing database pairs were created. We compared our proposed BWS algorithm and BLIINDS-II method on the same DCT domain. The SROCC results of the cross-database validation are tabulated in Table 17. The results indicate that the BWS algorithm performs better than the BLIINDS-II method in most cases. Therefore, the cross-database validation has demonstrated the generality and robustness of the proposed BWS method in DCT domain.

### 4.5. Discussion

#### 4.5.1. Model Selection

**Figure 10:**
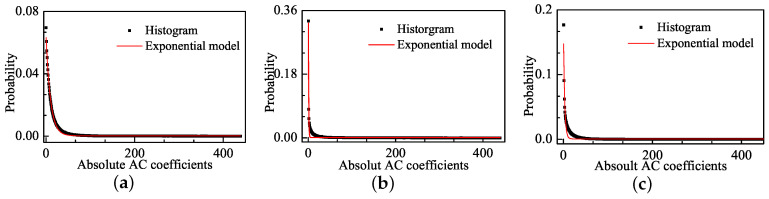
The distribution of absolute AC coefficients corresponding images and the Exponential fitting curves: (**a**) image with DMOS = 0; (**b**) image with DMOS = 63.649; and (**c**) image with DMOS = 29.739.

In Section 2, we analyze Weibull model as NSS model instead of a typical GGD model can well simulate the statistical regularities of distorted natural images in DCT domain. However, the changes of Weibull distribution model are similar to the simpler exponential distribution model. It is necessary to judge whether the exponential model is superior to Weibull model for IQA. Figure 10 shows the distribution of AC coefficients for the corresponding images in Figure 1 and the exponential fitting curve. We found that, if we used the exponential distribution model to fit statistics of natural and distorted images in DCT domain, it unfortunately failed at fitting these phenomena. Moreover, we used MSE to present fitting error comparison of three models for each distortion type images, as shown in Table 18. The fitting errors of Weibull model is minimum. Therefore, it is not appropriate to use exponential model instead of Weibull model.

#### 4.5.2. The Block Size Selection

In the BWS method, we selected 5×5 block for DCT. On the one hand, if a smaller block size is selected, the correlation between blocks is very large, thus it is difficult to distinguish the difference of extracted features. It affects the prediction accuracy. Similarly, if a bigger block size is selected, the extracted features lack the correlation information between blocks, which leads to inaccurate evaluation performance. Meanwhile, we used experiments to prove 5×5 block size is better in our method, as shown in Table 19. On the other hand, the 5×5 block is very common in image quality assessment [29,61,62]. Therefore, selecting 5×5 block is reasonable and can improve predicted performance.

#### 4.5.3. The Pooling Strategy and Multi-Scales Selection

Pooling strategy has been studied recently as an important factor to the accuracy of objective quality metrics. In our method, we calculated the average value of the highest 10% of features and that of 100% of features. The highest 10% and the 100% features of all blocks in image describe image distortion of the worst 10% regions and the overall image regions. The averaging of features in all the local regions is one of the widely used methods in image quality metrics. It describes the global distortion of the image. However, when only a small region in an image is corrupted with extremely annoying artifacts, human subjects tend to pay more attention to the low-quality region. Thus, we also considered the image distortion of the worst 10% regions because the human eye is more annoyed by the local distortion of the image and the subjects are likely to focus their quality opinions on the worst 10% of the whole image [63].

We extracted three feature sets: the average values of the highest 10% features, the average values of the 100% features and the combination of the first two feature sets in three scales from the LIVE database. The feature extraction method and the experimental setup were the same as those reported in Section 3 and Section 4. The median SROCC value of 1000 training-testing trials was utilized to evaluate the performance. The results of this experiment are shown in Table 20, and they indicate that the performance of the combination feature set is better than that when only one factor is considered. This finding demonstrates that the joint pooling strategy can improve the prediction performance of image quality measures and human intuition of images is the synthesis of local and global perception.

A multi-scale segmentation method has been developed and implemented for IQA. We proposed multi-scale features for predicting image quality because the perceptibility of image details depends on the viewing conditions. The subjective evaluation of a given image varies with these factors. Therefore, a multi-scale method is an effective method of incorporating image details at different resolutions.

We conducted an experiment to determine the impact of scale on IQA. In our method, we selected three scale images for extracting features because no significant gain in performance is obtained beyond the third scale of feature extraction. The methods of scale image segmentation, feature extraction and experiment setup were the same as the previous operation. We report the correlations of different scales between the predicted scores and the subjective scores on the LIVE database in Table 21. The experimental results show that the performance of BWS method in three scales outperform the other cases. It proves that the approach of multi-scale can improve the performance of our metric.

### 4.6. Computational Complexity

In many practical applications, the prediction accuracy and the algorithm complexity need to be considered comprehensively. Therefore, we evaluated the computational complexity of all competing methods in Table 22. One can see that, although the proposed BWS algorithm does not have the lowest complexity, the assessment accuracy is better among all the competing models, as shown in Table 10 and Table 16. For example, the BIQI is the lowest with complexity (N), where N is the total number of image pixels. However, its performance is the worst. The DIIVINE is the most complex, but the performance is inferior to our algorithm. Therefore, the proposed BWS algorithm is a reasonable trade-off between assessment accuracy and computational complexity.

## 5. Conclusion

Since there exists a strong relationship between the changes of entropy and distribution of images, existing NR IQA models typically use the GGD model to fit the distribution of a natural image in the DCT domain and extract features from this model to learn a quality prediction model. However, the difference between the distribution of the distorted image and that of its natural image, which includes the pulse-shape phenomenon, is neglected. In this paper, we propose the BWS method, which is a new NR IQA method based on the Weibull model in the DCT domain. The most important pulse-shape phenomenon is first considered in the distorted image distribution. The proposed Weibull model not only overcomes the disadvantages of the GGD model but also reflects HVS perception. Our research findings suggest that the Weibull model plays an important role in quality assessment tasks. Extensive experimental results on three public databases have demonstrated that the proposed BWS method is highly correlated with human visual perception and competitive with the state-of-the-art NR and FR IQA methods in terms of prediction accuracy and database independence.

## Figures and Tables

**Table 1:** The average MSE of each distortion type.

Types	Weibull	GGD
JP2K	$4.39\times 10^{-6}$	$2.51\times 10^{-4}$
JPEG	$4.79\times 10^{-6}$	$2.73\times 10^{-4}$
WN	$1.06\times 10^{-6}$	$1.19\times 10^{-5}$
GB	$2.29\times 10^{-6}$	$1.30\times 10^{-3}$
FF	$2.88\times 10^{-6}$	$5.73\times 10^{-4}$

**Table 2:** SROCC correlation of DMOS vs. ζ.

LIVE Subset	10%ζ	100%ζ
JP2K	0.824	0.772
JPEG	0.812	0.819
WN	0.981	0.988
GB	0.926	0.882
FF	0.760	0.786

**Table 3:** SROCC correlation of DMOS vs. ξ.

LIVE Subset	10%ξ	100%ξ
JP2K	0.922	0.899
JPEG	0.799	0.047
WN	0.961	0.937
GB	0.933	0.862
FF	0.832	0.843

**Table 4:** DCT coefficients of three bands.

DC	$C_{12}$	$C_{13}$	$C_{14}$	$C_{15}$
$C_{21}$	$C_{22}$	$C_{23}$	$C_{24}$	$C_{25}$
$C_{31}$	$C_{32}$	$C_{33}$	$C_{34}$	$C_{35}$
$C_{41}$	$C_{42}$	$C_{43}$	$C_{44}$	$C_{45}$
$C_{51}$	$C_{52}$	$C_{53}$	$C_{54}$	$C_{55}$

**Table 5:** SROCC correlation of DMOS vs. *f*.

LIVE Subset	10%*f*	100%*f*
JP2K	0.804	0.887
JPEG	0.845	0.862
WN	0.935	0.916
GB	0.713	0.821
FF	0.801	0.869

**Table 6:** DCT coefficient collected along three orientations.

DC	$C_{12}$	$C_{13}$	$C_{14}$	$C_{15}$
$C_{21}$	$C_{22}$	$C_{23}$	$C_{24}$	$C_{25}$
$C_{31}$	$C_{32}$	$C_{33}$	$C_{34}$	$C_{35}$
$C_{41}$	$C_{42}$	$C_{43}$	$C_{44}$	$C_{45}$
$C_{51}$	$C_{52}$	$C_{53}$	$C_{54}$	$C_{55}$

**Table 7:** SROCC correlations of DMOS vs. $S_{o}$.

LIVE Subset	10%$S_{o}$	100%$S_{o}$
JP2K	0.887	0.903
JPEG	0.813	0.748
WN	0.954	0.951
GB	0.928	0.923
FF	0.865	0.866

**Table 8:** Features used for BWS algorithm.

Scale	Feature Set
The first scale	$\left\{10\%\zeta ,100\%\zeta ,10\%\xi ,100\%\xi ,10\%f,100\%f,10\%o,100\%S_{o}\right\}$
The second scale	$\left\{10\%\zeta ,100\%\zeta ,10\%\xi ,100\%\xi ,10\%f,100\%f,10\%o,100\%S_{o}\right\}$
The third scale	$\left\{10\%\zeta ,100\%\zeta ,10\%\xi ,100\%\xi ,10\%f,100\%f,10\%o,100\%S_{o}\right\}$

**Table 9:** Overall performance on three databases.

Algorithms	LIVE		TID2008		CSIQ	
	SROCC	PLCC	SROCC	PLCC	SROCC	PLCC
PSNR	0.867	0.859	0.877	0.863	0.905	0.904
SSIM	0.913	0.907	0.780	0.755	0.834	0.835
BIQI	0.819	0.821	0.803	0.852	0.905	0.892
DIIVINE	0.912	0.917	0.898	0.893	0.878	0.896
BLIINDS-II	0.931	0.930	0.889	**0.916**	**0.911**	0.926
BRISQUE	**0.940**	**0.942**	**0.906**	0.914	0.902	**0.927**
BWS	**0.934**	**0.943**	**0.921**	**0.942**	**0.931**	**0.934**

**Table 10:** Performance of weighted average over three databases.

Algorithms	Weighted Average	
	SROCC	PLCC
PSNR	0.882	0.875
SSIM	0.914	0.916
BIQI	0.845	0.852
DIIVINE	0.897	0.905
BLIINDS-II	0.915	0.926
BRISQUE	0.920	0.931
BWS	**0.930**	**0.940**

**Table 11:** Statistical significance test on LIVE.

LIVE	BWS	BLIINDS-II	SSIM	PSNR
BWS	0	1	1	1
BLIINDS-II	−1	0	1	1
SSIM	−1	−1	0	1
PSNR	−1	−1	−1	0

**Table 12:** Statistical significance test on TID2008.

TID2008	BWS	BLIINDS-II	SSIM	PSNR
BWS	0	1	1	1
BLIINDS-II	−1	0	1	1
SSIM	−1	−1	0	1
PSNR	−1	−1	−1	0

**Table 13:** Statistical significance test on CSIQ.

CSIQ	BWS	BLIINDS-II	SSIM	PSNR
BWS	0	1	1	1
BLIINDS-II	−1	0	1	1
SSIM	−1	−1	0	1
PSNR	−1	−1	−1	0

**Table 14:** The SROCC comparison on individual distortion types.

Databases	Types	PSNR	SSIM	BIQI	DIIVINE	BLIINDS-II	BRISQUE	BWS
LIVE	JP2K	0.865	0.939	0.856	**0.932**	0.928	0.914	**0.929**
	JPEG	0.883	0.947	0.786	**0.948**	0.942	**0.965**	0.895
	WN	0.941	0.964	0.932	**0.982**	0.969	**0.979**	0.976
	GB	0.752	0.905	0.911	0.921	0.923	**0.951**	**0.932**
	FF	0.874	0.939	0.763	0.871	**0.889**	0.877	**0.893**
TID2008	JP2K	0.854	0.900	0.857	0.826	**0.922**	0.895	**0.933**
	JPEG	0.886	0.931	0.859	0.913	**0.918**	0.910	**0.914**
	WN	0.923	0.836	0.798	**0.896**	0.805	0.862	**0.868**
	GB	0.944	0.954	**0.901**	**0.901**	0.868	0.890	0.892
CSIQ	JP2K	0.942	0.929	0.901	0.904	0.900	**0.951**	**0.912**
	JPEG	0.893	0.934	0.906	0.879	0.920	**0.925**	**0.922**
	WN	0.938	0.936	**0.921**	0.897	0.913	0.878	**0.937**
	GB	0.940	0.906	0.874	0.866	**0.941**	0.902	**0.954**

**Table 15:** The PLCC comparison on individual distortion types.

Databases	Types	PSNR	SSIM	BIQI	DIIVINE	BLIINDS-II	BRISQUE	BWS
LIVE	JP2K	0.876	0.941	0.809	0.922	**0.935**	0.923	**0.945**
	JPEG	0.903	0.946	0.901	0.921	**0.968**	**0.973**	0.923
	WN	0.917	0.982	0.939	**0.988**	0.980	**0.985**	0.984
	GB	0.780	0.900	0.829	0.923	0.938	**0.951**	**0.952**
	FF	0.880	0.951	0.733	0.868	0.896	**0.903**	**0.903**
TID2008	JP2K	0.906	0.906	0.891	0.810	**0.946**	0.905	**0.957**
	JPEG	0.896	0.961	0.883	0.906	**0.952**	0.923	**0.961**
	WN	0.953	0.852	0.823	**0.908**	0.840	0.862	**0.893**
	GB	0.950	0.955	**0.929**	0.898	0.906	0.896	**0.909**
CSIQ	JP2K	0.950	0.943	0.897	0.918	0.930	**0.957**	**0.938**
	JPEG	0.905	0.958	0.884	0.896	0.931	**0.956**	**0.940**
	WN	0.952	0.940	**0.929**	0.921	0.917	0.906	**0.942**
	GB	0.959	0.913	0.875	0.887	**0.926**	0.920	**0.944**

**Table 16:** Performance of weighted average and STD across all distortion groups.

Algorithms	Weighted Average		Weighted STD	
	SROCC	PLCC	SROCC	PLCC
PSNR	0.894	0.908	0.188	0.169
SSIM	**0.927**	**0.937**	**0.113**	**0.118**
BIQI	0.867	0.871	0.205	0.207
DIIVINE	0.906	0.908	0.137	0.139
BLIINDS-II	0.915	0.930	0.141	0.122
BRISQUE	0.919	0.932	0.128	0.122
BWS	**0.922**	**0.938**	**0.099**	**0.087**

**Table 17:** SROCC comparison on cross-database validation.

Train	Test	BWS	BLIINDS-II
LIVE	TID2008	**0.889**	0.844
LIVE	CISQ	0.839	0.868
TID2008	LIVE	**0.867**	0.742
TID2008	CISQ	0.828	0.853
CISQ	LIVE	**0.884**	0.833
CISQ	TID2008	**0.869**	0.832

**Table 18:** The average MSE of each distortion type.

Types	Weibull	GGD	Exponential
JP2K	$4.39\times 10^{-6}$	$2.51\times 10^{-4}$	$1.32\times 10^{-4}$
JPEG	$4.79\times 10^{-6}$	$2.73\times 10^{-4}$	$1.09\times 10^{-4}$
WN	$1.06\times 10^{-6}$	$1.19\times 10^{-5}$	$1.21\times 10^{-4}$
GB	$2.29\times 10^{-6}$	$1.30\times 10^{-3}$	$1.41\times 10^{-4}$
FF	$2.88\times 10^{-6}$	$5.73\times 10^{-4}$	$1.31\times 10^{-4}$

**Table 19:** SROCC correlation of DMOS vs. 100%ζ.

LIVE Subset	3×3	5×5	8×8
JP2K	0.719	**0.772**	0.702
JPEG	0.801	**0.819**	0.768
WN	0.969	**0.988**	0.976
GB	0.865	**0.882**	0.875
FF	0.754	**0.768**	0.732

**Table 20:** SROCC performance with pooling strategy.

Types	Highest 10% Set	100% Set	Combination Set
JP2K	0.900	0.915	**0.929**
JPEG	0.850	0.872	**0.895**
WN	0.975	0.974	**0.976**
GB	0.906	0.921	**0.932**
FF	0.851	0.878	**0.893**
ALL	0.901	0.913	**0.934**

**Table 21:** SROCC performance with multi-scale selection.

Types	One Scale	Two Scales	Three Scales
JP2K	0.905	0.926	**0.929**
JPEG	0.857	0.877	**0.895**
WN	0.973	**0.979**	0.976
GB	0.923	0.928	**0.932**
FF	0.837	0.864	**0.893**
ALL	0.904	0.921	**0.934**

**Table 22:** Computational complexity. *N* is the total number of pixels in a test image.

Algorithms	Computational Complexity	Notes
BIQI	O(N)	
DIIVINE	$O(Nlog\left(N\right)+m^{2}+N+392b)$	m:neighborhood size in DNT; b:2D histogram bin number
BRISQUE	$O(d^{2}N)$	d:block size
BLIINDS-II	$O(\left(N/d^{2}\right)log\left(N/d^{2}\right))$	d:block size
BWS	$O(\left(N/d^{2}\right)log\left(N/d^{2}\right))$	d:block size

## Abbreviations

The following abbreviations are used in this manuscript:

FR	Full-Reference
RR	Reduced-Reference
NR	No-Reference
IQA	Image Quality Assessment
NSS	Natural Scene Statistics
BWS	Blind Weibull Statistics
DCT	Discrete Cosine Transform
AC	Alternating Current
HVS	Human Visual System
SVR	Support Vector Regression
GGD	Generalized Gaussian Distribution
DMOS	Difference Mean Opinion Score
MSE	Mean Square Error
JP2K	JP2K compression
JPEG	JPEG compression
WN	White Noise
GB	Gaussian Blur
FF	Fast Fading
MOS	Mean Opinion Score
SROCC	Spearman Rank Order Correlation
PLCC	Pearson Linear Correlation Coefficient
STDs	Standard Deviations
PSNR	Peak Signal to Noise Ratio
